# Mating systems and predictors of relative reproductive success in a Cutthroat Trout subspecies of conservation concern

**DOI:** 10.1002/ece3.7914

**Published:** 2021-07-24

**Authors:** John S. Hargrove, Jesse McCane, Curtis J. Roth, Brett High, Matthew R. Campbell

**Affiliations:** ^1^ Pacific States Marine Fisheries Commission Eagle ID USA; ^2^ Idaho Department of Fish and Game Nampa ID USA; ^3^ Idaho Department of Fish and Game Idaho Falls ID USA; ^4^ Idaho Department of Fish and Game Eagle ID USA

**Keywords:** effective population size, *Oncorhynchus clarkii bouvieri*, parentage‐based tagging, sibship analysis, Yellowstone Cutthroat Trout

## Abstract

Mating systems and patterns of reproductive success in fishes play an important role in ecology and evolution. While information on the reproductive ecology of many anadromous salmonids (*Oncorhynchus* spp.) is well detailed, there is less information for nonanadromous species including the Yellowstone Cutthroat Trout (*O. clarkii bouvieri*), a subspecies of recreational angling importance and conservation concern. Using data from a parentage‐based tagging study, we described the genetic mating system of a migratory population of Yellowstone Cutthroat Trout, tested for evidence of sexual selection, and identified predictors of mating and reproductive success. The standardized variance in mating success (i.e., opportunity for sexual selection) was significantly greater for males relative to females, and while the relationship between mating success and reproductive success (i.e., Bateman gradient) was significantly positive for both sexes, a greater proportion of reproductive success was explained by mating success for males (*r* ^2^ = 0.80) than females (*r* ^2^ = 0.59). Overall, the population displayed a polygynandrous mating system, whereby both sexes experienced variation in mating success due to multiple mating, and sexual selection was variable across sexes. Tests for evidence of sexual selection indicated the interaction between mating success and total length best‐predicted relative reproductive success. We failed to detect a signal of inbreeding avoidance among breeding adults, but the group of parents that produced progeny were on average slightly less related than adults that did not produce progeny. Lastly, we estimated the effective number of breeders (*N*_b_) and effective population size (*N*_e_) and identified while *N*_b_ was lower than *N*_e_, both are sufficiently high to suggest Yellowstone Cutthroat Trout in Burns Creek represent a genetically stable and diverse population.

## Graphical abstract

We collected genetic samples from over 1,500 adult Yellowstone Cutthroat Trout as they entered a spawning tributary and subsequently collected ~3,000 genetic samples from outmigrating juveniles from the same creek system. Using a combination of sibship analysis and parentage‐based tagging, we identified a polygynandrous mating system, whereby both sexes experienced variation in mating success due to multiple mating, and sexual selection was variable across sexes. These data have direct implications for ongoing management and conservation efforts.

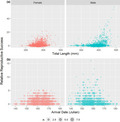

## 1 INTRODUCTION

Freshwater fishes display a diversity of mating systems and reproductive behaviors (DeWoody & Avise, 2001), and knowledge of reproductive ecology and drivers of reproductive success are relevant to both conservation and management efforts. For example, effective population size (*N*_e_) measures the rate of genetic drift within a population and can be influenced by population structure (e.g., discrete vs. overlapping generations), changes in population size, and the strength of sexual selection (Charlesworth, 2009). Populations that exhibit large variances in reproductive success, unequal family sizes, and heavily skewed sex ratios will have lower values of *N*_e_, experience losses in genetic diversity via genetic drift, and be at an elevated risk of extirpation relative to idealized populations (e.g., equal family size, sex ratios, and low variance in reproductive success; Frankham, 2005). In addition to potentially impacting persistence, mating systems and knowledge thereof may be directly applicable to hatchery supplementation efforts whereby managers may wish to emulate patterns observed in the wild.

Pacific salmonids (*Oncorhynchus* spp.) are of profound recreational, ecological, and economic importance throughout their range (Quinn, 2018). Rates of imperilment among salmon are high (Gustafson et al., 2007; Muhlfeld et al., 2019), and efforts to assess the viability of distinct population segments include quantifying levels of genetic diversity (McElhany et al., 2000). Given the direct link between reproductive characteristics and levels of genetic diversity, an understanding of sexual selection mechanisms (including strength and direction) driving mating success and by extension variation in reproductive success is directly relevant to conservation planning and management. The mating systems and predictors of reproductive success of many salmonid species have been described in detail (Brook Trout, *Salvelinus fontinalis*: Kanno et al., 2011; Steelhead, *Oncorhynchus mykiss*: Seamons et al., 2004a; McMillan et al., 2007; Atlantic salmon, *Salmo salar*: Garant et al., 2001); however, there are no such data for subspecies such as Yellowstone Cutthroat Trout (*Oncorhynchus clarkii bouvieri*).

Yellowstone Cutthroat Trout are an iconic species of the western United States and are one of three subspecies of Cutthroat Trout native to Idaho. Yellowstone Cutthroat Trout are thought to occupy less than half of their historical habitats rangewide (May et al., 2003; Meyer et al., 2006) and are considered to be a high‐priority species with a standalone management and conservation plan (IDFG, 2007). Primary factors associated with declines include hybridization with non‐native Rainbow Trout (*O. mykiss*), competition with invasive species (e.g., Brook Trout), overharvest, water diversion, and habitat alterations (Behnke, 1992; Kruse et al., 2000; Varley & Gresswell, 1988). Despite a range contraction relative to historical records, the abundance and size structure of Yellowstone Cutthroat Trout in Idaho have remained stable over the last 20 years (Meyer et al., 2003, 2014). Although Yellowstone Cutthroat Trout have been extensively studied, we found no published literature describing their mating systems. Recent research tested if angling impacts the reproductive success of Yellowstone Cutthroat Trout (Roth et al., 2018), and the associated data sets have afforded the opportunity to use extensive field sampling of parents and progeny to describe mating systems and patterns of reproductive success.

We used an existing data set to describe the genetic mating system of Yellowstone Cutthroat Trout. Specifically, we made use of data collected by Roth et al. (2018) in which migratory adults were sampled at a weir as they entered a spawning tributary as well as their outmigrating juvenile offspring as they left the system. All sampled adults and juveniles were genotyped using a panel of single nucleotide polymorphisms (SNPs), and a combination of parentage analysis and sibship estimation (i.e., identifying sibling relationships among a sample of offspring using multilocus genotype data) was used to estimate the mating success and reproductive success of all sampled parents. These data were used to describe population‐level means and variances in mating success and reproductive success for adult males and females and to model the relationship between mating success and reproductive success (i.e., Bateman's principle; Bateman, 1948). Additionally, we tested for the presence of sexual selection on phenotypic traits (total length and arrival timing at spawning grounds) and modeled how these factors along with mating success predicted reproductive success. Lastly, we tested for evidence of inbreeding avoidance among mating adults and estimated the effective population size (*N*_e_) and effective number of breeders (*N*_b_) of Yellowstone Cutthroat Trout in Burns Creek. Combined, we present the first detailed description of the mating patterns of each sex and the genetic mating system of a Yellowstone Cutthroat Trout population and the sexually selected traits influencing mating and reproductive success.

## 2 METHODS

### 2.1 Sample collection

The samples used in the current study were a subset of those described in Roth et al. (2018) which assessed the effects of air exposure and angling on short‐ and long‐term survival as well as progeny production of adult Yellowstone Cutthroat Trout. Results identified air exposure had no statistically significant effect on the proportion of fish that successfully spawned and neither angling nor air exposure significantly affected progeny production (Roth et al., 2018). Given the lack of observed impacts of air exposure and angling on reproductive contributions and reproductive success, we concluded data on mating systems for Yellowstone Cutthroat Trout derived from the samples of Roth et al. (2018) would be generally reflective of patterns in nature.

**FIGURE 1:**
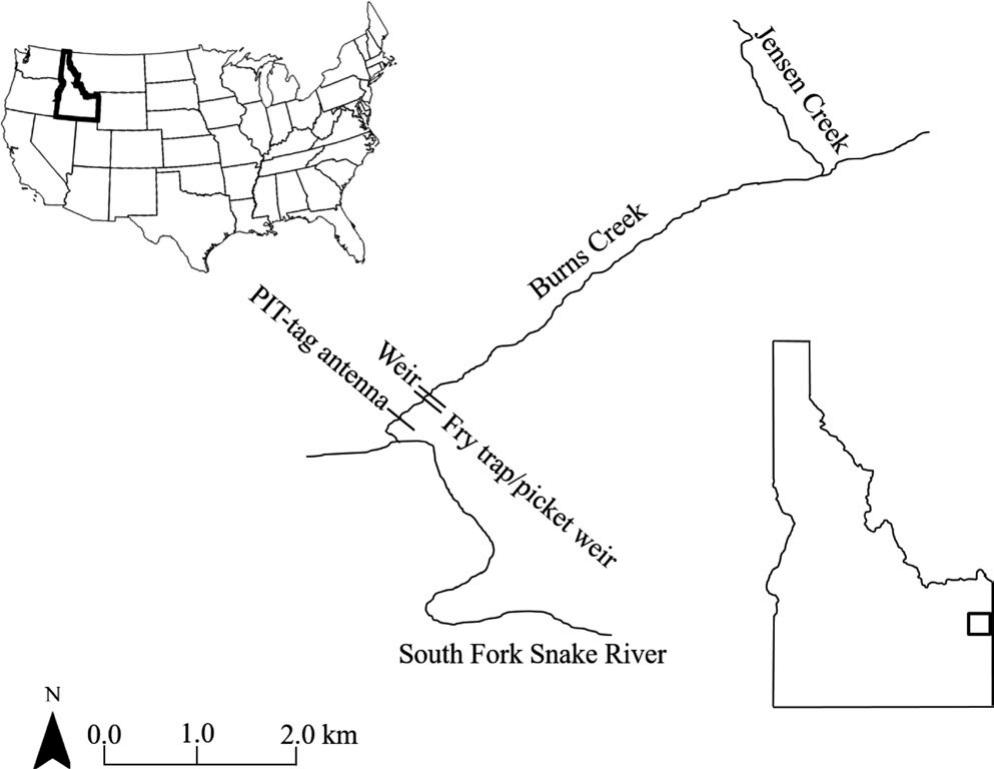
A map of Burns Creek, a tributary to the South Fork Snake River in Idaho. Marked on Burns Creek is the placement of the adult weir (labeled “weir”) where upstream‐migrating Yellowstone Cutthroat Trout (*Oncorhynchus clarkii bouvieri*) were sampled for genetics. Outmigrating juveniles were sampled at the picket weir and a Kray–Meekin fry trap

A detailed description of all sampling methods can be found in Roth et al. (2018). Briefly, sampling was conducted on Burns Creek, Idaho (Figure 1), a tributary to the South Fork Snake River, from May to October 2016. Discharge in Burns Creek typically varies from 0.1 to 9.0 m^3^/s and channel gradient is 3%–6%. A large portion of Yellowstone Cutthroat Trout in the South Fork Snake River (SFSR) displays a fluvial life‐history strategy with adults migrating from the mainstem into tributary systems to spawn (Thurow et al., 1988). Yellowstone Cutthroat Trout in the SFSR mature around age 4 and spawning begins in late May and continues through early July. Fluvial Yellowstone Cutthroat Trout generally spawn in third‐order streams in the Burns Creek drainage. This includes 9.5 river km of stream in Burns Creek and Little Burns Creek. Resident life‐history forms of YCT are found in an additional 12.6 river km of streams including the uppers sections of Burns Creek and three tributaries. Approximately 2 weeks after spawning, adults migrate from Burns Creek back to the mainstem SFSR. Fry in Burns Creek emerge between mid‐July and September and outmigrate to the mainstem river as age‐0 fish (Moore & Schill, 1984; Thurow et al., 1988).

Adult Yellowstone Cutthroat Trout were sampled from May through July 2016 at an adult weir operated on Burns Creek, 0.9 km upstream of the creek mouth. The timing of sampling was geared to capture the entire spawning migration by Yellowstone Cutthroat Trout into Burns Creek. Fish migrating upstream into Burns Creek enter a fish trap located at the top of a fish ladder which is utilized to reach habitats upstream from the weir. The adult trap is operated annually by the IDFG with the purpose of removing straying Rainbow Trout and hybrids. A genetic fin clip was taken from each migrating adult and stored on Whatman 3MM chromatography paper (Thermo Fisher Scientific, Inc.). The phenotypic sex of each adult was identified in the field (which included checking for the expression of milt or presence of ovipositor) and confirmed using a genetic assay which is highly accurate (99%; Schill et al., 2016).

Because sexually immature fish included among adult samples can introduce bias into tests of sexual selection, we screened our pool of potential parents to remove fish that failed to mate because they were not sexually mature. To this end, we dropped adults without a phenotypic sex call from the analysis (*n* = 16). Additionally, we recorded total length for each adult and used maturity schedules for nearby, migratory populations of Yellowstone Cutthroat Trout to exclude immature fish. Specifically, Meyer et al. (2003) presented length at 50% maturity (L_50_) estimates for 3 nearby populations of migratory Yellowstone Cutthroat Trout (mean L_50_ for females = 219.0 mm; males = 192.4 mm), and we omitted all adult females less than 250 mm in length (*n* = 2) and males less than 200 mm (*n* = 1) using these data. Lastly, because migrations to natal spawning areas are energetically costly, the likelihood of sexually immature fish undergoing a spawning migration was thought to be low.

Outmigrating age‐0 Yellowstone Cutthroat Trout were collected later the same year using a combination of trapping and electrofishing methods. Fry were collected using a modified picket weir approximately 25 m downstream of the adult weir/trap (described above) as well as with one Kray–Meekin trap placed in the thalweg downstream of the weir (Figure 1). Trapping occurred continuously between July and October in 2016. Additionally, single‐pass backpack electrofishing was used to supplement fry collection and was performed over two 2‐d periods in September and October. Backpack electrofishing was performed from the IDFG picket weir upstream for 4 km as this section represents the area where the majority of fluvial Yellowstone Cutthroat Trout spawn (Brett High, Idaho Department of Fish and Game, unpublished data). A subset of fry collected via traps and electrofishing were randomly sampled for genetic tissue. We assumed the cumulative fry sampling was an accurate representation of total production/ outmigration and made every effort to minimize sampling bias that may have arisen due to sampling error.

### 2.2 Genetic analysis

Genomic DNA was extracted from fin clips using the nexttec Genomic DNA Isolation Kit (XpressBio, Thurmont, Maryland) following the manufacturer's protocol. Samples were screened with a panel of 134 single nucleotide polymorphic (SNP) loci which included a sex‐linked marker (*2017SDYCUT*) used to identify genetic sex of each fish (see Roth et al., 2018 for sex‐linked primer details). Genetic sex data were used to verify parentage assignments were attributed to a male and female combination. Primer sequences for all SNPs are available from the authors upon request. Genotyping of SNPs was performed following the genotyping‐in‐thousands protocol described by Campbell et al. (2015). We screened all genotypes for duplicates and completeness; only unique genotypes ≥50% complete (minimum of 66 loci) were retained for analyses.

The nonlethal genetic tagging method known as parentage‐based tagging (PBT) was used to identify parent pair offspring relationships among sets of adult and offspring samples based on patterns in Mendelian inheritance (Steele et al., 2013, 2019). Results from PBT were then used to characterize the genetic mating systems of a Yellowstone Cutthroat Trout population by estimating the means and variances in mating success and reproductive success within and between sexes (see below).

Parentage‐based tagging was applied by Roth et al. (2018) to identify parent pair–offspring relationships. Specifically, adults were exhaustively sampled during their migration to spawning grounds, and SNP genotype profiles were created for each adult. With this approach, we effectively genetically tagged all offspring that would be the byproduct of matings among sampled adults. Parentage assignments in trios (both parents and one offspring) were estimated using the likelihood approach implemented within SNPPIT (Anderson, 2010; Anderson & Garza, 2005) assuming a per‐allele genotyping error rate of 0.5%. The confidence of assignments was assessed using the logarithm of odds (LOD) score generated by SNPPIT. The LOD score represents the natural logarithm of the likelihood of the parental trio hypothesis divided by the likelihood of the unrelated hypothesis for a trio. Only parentage assignments with a LOD score ≥18 were retained as previous analysis has identified that this threshold minimized false‐positive and false‐negative assignments (Roth et al., 2018).

Two forms of parentage error, type I and II error, were quantified by Roth et al. (2018) to assess the reliability of parentage assignments for this data set. Type I error measures the rate that untrue parent pairs were assigned as a true parent, and Type II error refers to the number of offspring for which a true parent pair was present but not assigned. Type I error was estimated using two separate methods and was determined to be 0.000 and 0.001. Type II error was estimated to be 0.001.

Although we expected the incidence of false‐positive and false‐negative rates for parentage assignment to be low because we used stringent criteria to limit error (see above), we elected to use a complimentary analysis, sibship assignment (SA), to confirm PBT results. Briefly, we estimated full‐sibling relationships among our juvenile collection using the sibship assignment approach implemented in the software program COLONY (Jones & Wang, 2010) and compared the outputs from SA and parentage analysis. COLONY employs a full‐likelihood approach to estimate sibship and outperforms other sibship reconstruction methods (Lepais et al., 2010). For comparison purposes, we determined the proportion of full‐sibling pairs that were identical based on the two estimation procedures. Additionally, we examined the number of full‐sibling pairs detected by one program and not the other. Lastly, we report on the number of single children families detected via sibship assignment and parentage analysis.

The discriminatory power of the 133 SNP loci used for parentage analysis was evaluated by calculating exclusion probabilities, which represent the probability an unrelated candidate parent will be eliminated from consideration by the locus in question (Jones et al., 2010). We generated three probabilities of paternity exclusion in GenAlEx v 6.5 (Peakall & Smouse, 2006); (a) the probability of excluding a parent when one parent is known, (b) the probability of excluding a parent when the genotype of one parent is missing, and (c) the probability of excluding a putative parent pair.

### 2.3 Relative reproductive success, mating success, and adult sex ratio

Outputs from parentage analysis were used to generate individual profiles for each adult which included the number of offspring produced (relative reproductive success, see below), the number of unique mates (mating success), and the number of offspring produced per mate. Mating success was calculated as the total number of mates with whom an individual produced offspring. Because Roth et al. (2018) did not quantify total offspring production (i.e., only a subset of offspring were analyzed for genetic analysis), we present measures of relative reproductive success among adults based on the number of analyzed offspring assigned to a given parent.

Individual reproductive profiles were then used to estimate the sex‐specific means and variances of mating success and relative reproductive success. We tested for significant differences in mating success, relative reproductive success, and associated variances as a function of sex. First, we assessed the normality of mating success and relative reproductive success data using a Shapiro–Wilk test. Based on the observed deviations for normality (mating success: *W* = 0.73, *p* = 2.2 × 10^–16^; relative reproductive success *W* = 0.62, *p* = 2.2 × 10^–16^), mating success and relative reproductive success mean values were compared by sex using a Kruskal–Wallis test. Significant deviations in the distribution of variances by sex were assessed using Levene's *F* test (Mobley, 2014).

The opportunity for selection (*I*; Arnold & Wade, 1984) was calculated for each sex as the standardized variance in relative reproductive success (variance in relative reproductive success divided by the squared mean for the population; Arnold & Wade, 1984). The opportunity for sexual selection (*I*_s_) was calculated for each sex as the standardized variance in mating success (variance in mating success divided by the squared mean mating success for the population; Wade & Arnold, 1980). The “Bateman gradient,” or statistical relationship between mating success and relative reproductive success (Bateman, 1948) was fitted for males and females separately using a linear model. The adult sex ratio (ASR) was calculated as the proportion of adults in a population that are male, as it is a primary constraint on mating success (Schacht et al., 2017). All statistical tests were performed in R (R Core Team, 2020).

### 2.4 Sexual selection

We tested for evidence of sexual selection acting on phenotypic traits including arrival date at the weir (a proxy for arrival date at the spawning ground) and total length. First, we tested for differences in trait means for fish that did and did not mate using Kruskal–Wallis test. Tests were performed independently for males and females. Next, we estimated correlation coefficients between phenotypic trait values (arrival date, total length) and mating success and relative reproductive success using Pearson's correlation coefficient. Statistical tests and summaries were performed in R (R Core Team, 2020).

The effects of total length, arrival date, and mating success on relative reproductive success were modeled using generalized linear models for males and females, separately. A suite of twelve a priori models were considered: (a) a null model (intercept), (b) length (total length), (c) date (fitted as the quadratic function: arrival time * arrival time^2^), (d) length + date, (e) length * date (representing an interaction between length and date), (f) mating success, (g) mating success + length, (h) mating success * length, (i) mating success + date, (j) mating success * date, (k) mating success + length + date, and (l) mating success + length * date. We used generalized linear models with a negative binomial distribution to account for overdispersion in offspring production using the MASS package (Venables & Ripley, 2002) in R. Each model was evaluated separately for males and females, and models were compared using Akaike's information criterion corrected for small sample size (AIC*c*). Our top model was the one with the lowest AIC*c* value (Burnham & Anderson, 2002); however, we also considered models with an AIC*c* score within 2.0 units of the best model's score as belonging to the set of top models. Model fit was assessed using McFadden's pseudo‐*R* ^2^ (McFadden, 1974).

### 2.5 Inbreeding avoidance among spawning adults

We tested for the presence of inbreeding avoidance among adult Yellowstone Cutthroat Trout to understand whether there are mechanisms that prevent breeding among related individuals. To do this, we first evaluated the accuracy of four different relatedness estimators (Li et al., 1993; Lynch & Ritland, 1999; Queller & Goodnight, 1989; Wang, 2002) based on correlation coefficients between observed and expected values. We employed the “compareestimators” function in the *related* package in R (Pew et al., 2015; R Core Team, 2020) which used observed allele frequencies within our focal population to simulate 1,000 individuals of known relatedness and selected the estimator with the highest correlation coefficient between known and estimated relationships. Next, we used outputs from parentage analysis to identify two subsets of the adult population, adults responsible for producing progeny and those that were not. We then calculated average within‐group relatedness using the best‐fit relatedness estimator for these two groups and compared against expected values using the “grouprel” function within the *related* package. Briefly, expected values were generated by randomly shuffling individuals between groups (which were kept at a constant size), and then, relatedness was calculating within these newly constructed groups. We performed a total of 100 simulations, and *p*‐values were estimated as the number of simulations out of the total number ran that were less than or equal to our observed value.

### 2.6 Effective population size (*N*_e_) and number of breeders (*N*_b_)

Effective population size can be estimated on multiple different scales, including over a generation (*N*_e_) or single reproductive cycle (*N*_b_, effective number of breeders). Both metrics provide insights relevant to conservation and management. Namely, *N*_e_ quantifies the extent of drift and inbreeding experienced in a given population (Charlesworth, 2009) and *N*_b_ estimates the number of effective breeders for a single reproductive season. A number of factors affect both *N*_e_ and *N*_b_, including operational sex ratio, variation in family size, inbreeding, and changes in population size; however, these factors operate at different time scales (Waples, 2002). We estimated both *N*_e_ and *N*_b_ for Yellowstone Cutthroat Trout, using the full‐likelihood method implemented in COLONY assuming random mating. Effective population size was calculated using the pooled parental population, which represented multiple different age classes. In contrast, we estimated *N*_b_ using only outmigrating juveniles, which would estimate the number of breeders contributing to the juvenile cohort.

## 3 RESULTS

### 3.1 Sample collection and genetic analysis

In 2016, a total of 1,520 upstream‐migrating adult Yellowstone Cutthroat Trout and 2,925 fry were sampled for genetic tissue. Overall genotype success rates were very high, with 99.9% of adult samples (1,518) and 99.7% of the juvenile samples (2,917) successfully amplifying at 50% or more of the SNP marker panel. Searches for duplicate genotypes identified a total of 15 adult samples which were near‐exact matches (>95% similar) of another sample, which likely represented adults that passed above the weir, fell back, and were sampled again when they reascended the weir (<1% of samples). Duplicate searches among offspring identified seven samples which were a duplicate of another (0.24% of samples). For both adults and offspring, only one genotype was retained from each pair of duplicates, whichever was the most complete (i.e., had the largest number of alleles called). The probability of exclusion estimates for all three metrics was 1 at the full marker panel, indicating sufficient resolution to confidently resolve parentage assignments.

**FIGURE 2:**
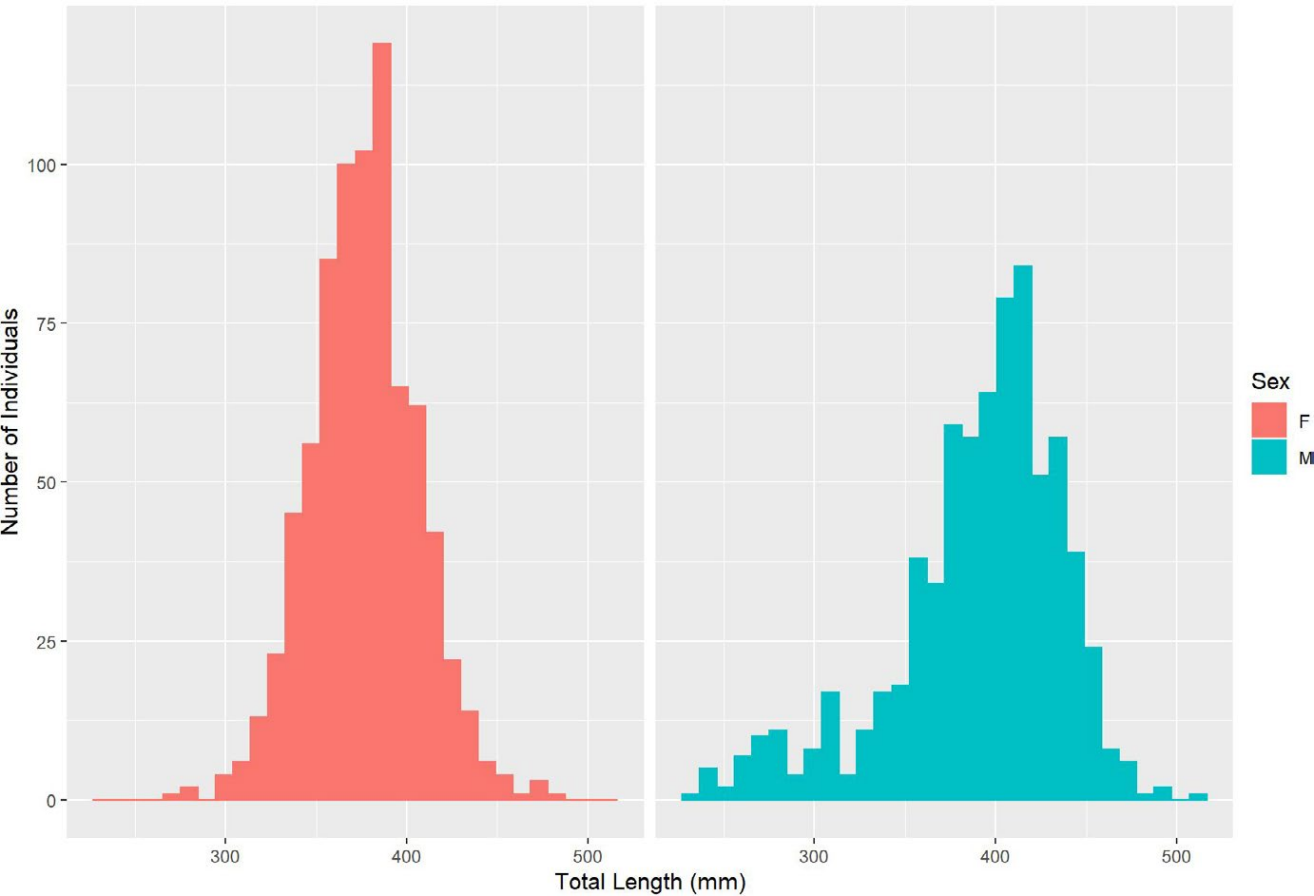
A length frequency histogram of migratory Yellowstone Cutthroat Trout (*Oncorhynchus clarkia bouvieri*) sampled at an instream weir on Burns Creek, Idaho, to detail genetic mating systems and identify predictors of reproductive success

After the removal of adults from the data set which lacked either phenotypic sex identification (16) or were potentially sexually immature based on length at maturity data (*n* = 3, see Methods for additional details), we had complete genotype profiles for 1,495 adults. The length of males ranged from 228 to 508 mm TL (average = 391 mm) and 267 to 485 (average = 377) for females (Figure 2). The number of adults male and females sampled was roughly equivalent (females = 776, males = 719), and the adult sex ratio (ASR) was 0.48.

Parentage analysis assigned 2,310 (78.7% of total number) of the fry samples to a parent pair handled at the weir. The same set of 3,862 full‐sibling pairs were identified by both sibship assignment and parentage analysis. Seventeen full‐sib pairs were detected by sibship assignment only, and 35 full‐sib pairs were detected exclusively by parentage analysis. Discordance between methods (52 pairs differed of 3,914 total pairs) was 1.3% indicating both methods produced highly similar results. Both analyses identified 410 “only children” (i.e., had no full‐siblings) offspring, and an additional 33 “only children” were detected by sibship assignment and not PBT. Three “only children” were detected by PBT but not sibship analysis.

### 3.2 Mating systems and reproductive success inferred via PBT

**FIGURE 3:**
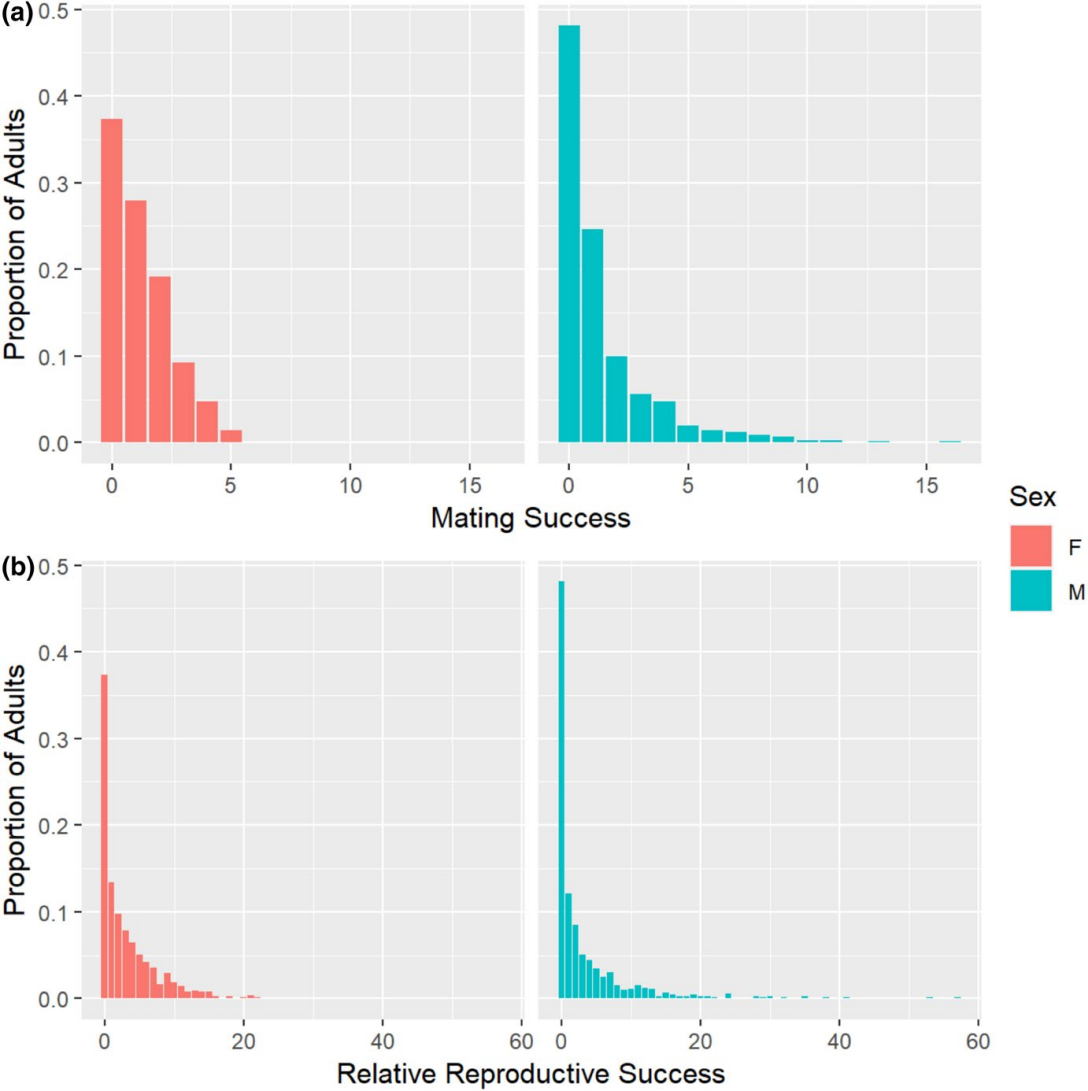
(a) A histogram of the number of mates acquired by female and male Yellowstone Cutthroat Trout (*Oncorhynchus clarkii bouvieri*) from Burns Creek, Idaho, during the 2016 spawning season. (b) A histogram of relative reproductive success as measured by the number of offspring assigned to each adult by sex

Based on parentage results, a total of 934 unique parent pairs were identified from a combination of 373 male and 486 female Yellowstone Cutthroat Trout. The number of mates per adult was right skewed, with no offspring contributions detected from 37.4% of females and 48.1% of males (Figure 3). Mean values of mating success were significantly greater for males (*µ* = 1.30) than females (*µ* = 1.20; *χ* ^2^ = 9.67, *df* = 1, *p*‐value = 0.002), with males mating with up to 16 different females. In contrast, females mated with up to five males (Figure 3). Mean relative reproductive success was significantly greater for males (*µ* = 3.21) than females (*µ* = 2.98; *χ* ^2^ = 11.67, *df* = 1, *p*‐value = <0.001; Table 1). The maximum number of offspring attributed to a female was 22, whereas one male produced 57 offspring.

**TABLE 1:** Estimates of mating success and relative reproductive success for a migratory population of Yellowstone Cutthroat Trout (*Oncorhynchus clarkii bouvieri*) sampled from Burns Creek, Idaho

	Females				Males				Kruskal–Wallis			Levene's test		
	*n*	Mean	*SE*	*I*_females_	*n*	Mean	*SE*	*I*_males_	*χ* ^2^	*df*	*P*	*F*	*df*	*p*
Relative reproductive success	776	2.98	0.14	10.5	719	3.21	0.23	23.2	11.67	1	<0.001	3.39	1	0.07
Mating success	776	1.20	0.04	1.07	719	1.30	0.08	2.45	9.67	1	0.002	22.22	1	2.6 × 10^–6^

Note

Sample sizes, mean values, standard errors (*SE*), and variances are presented for males and females. Differences in mean values and variances for mating success and relative reproductive success were estimated via Kruskal–Wallis and Levene's test, respectively. *p* values in italics were significant at *α* = 0.05 level. The opportunity for selection (*I*) represents the standardized variance in relative reproductive success whereas the opportunity for sexual selection represents the variance in mating success (*I*_s_).

Both the opportunity for selection (*I*) and opportunity for sexual selection (*I*_S_) were higher (≥2.2×) for males than for females (Table 1). Variances in mating success were significantly different as a function of sex (*F* = 22.22, *df* = 1, *p*‐value = 2.6 × 10^–6^); however, variances in relative reproductive success were not (*F* = 3.39, *df* = 1, *p*‐value = 0.07).

**FIGURE 4:**
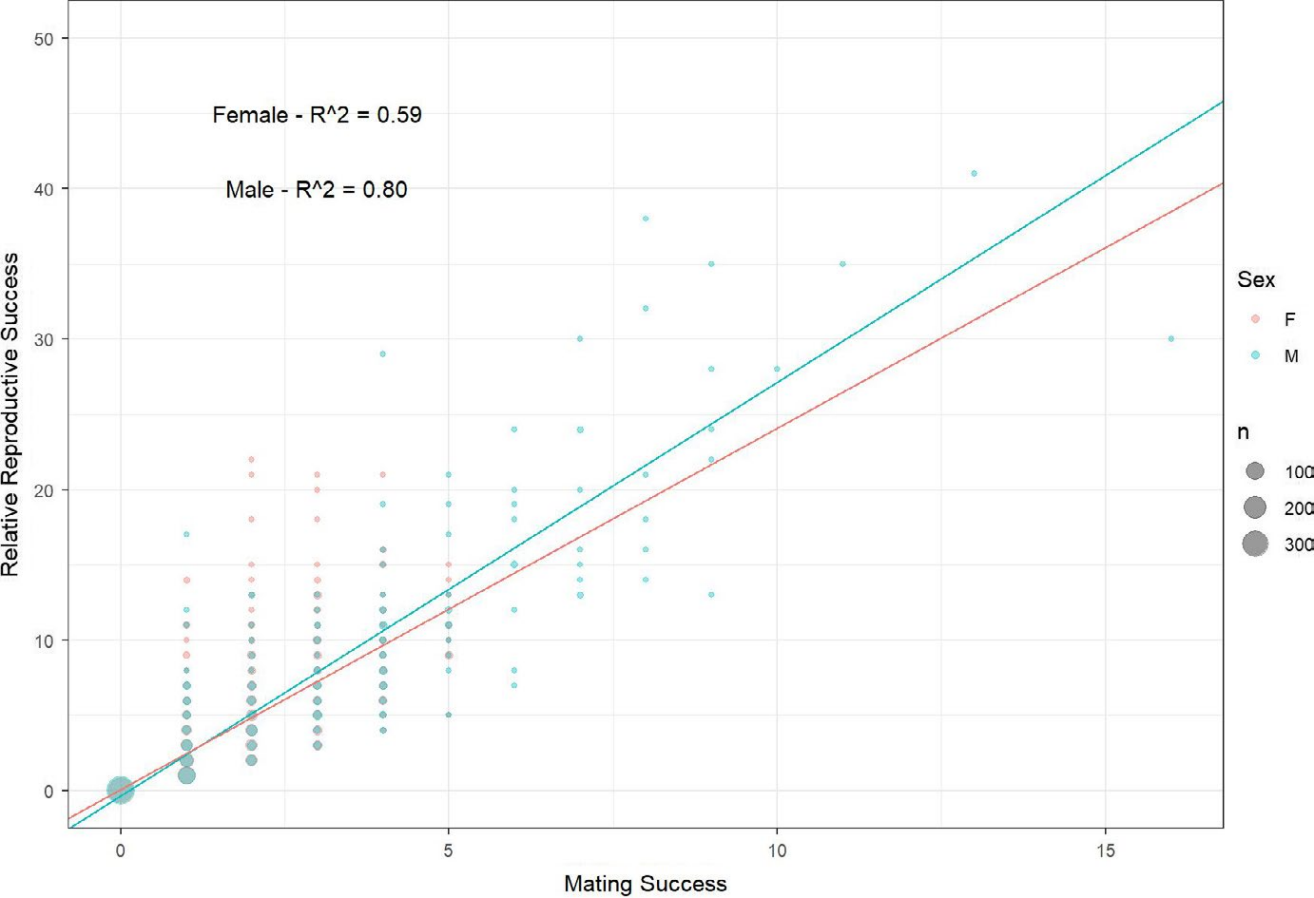
Bateman gradients for female (pink) and male (blue) Yellowstone Cutthroat Trout (*Oncorhynchus clarkii bouvieri*) showing sex‐specific relationships between the mating success and relative reproductive success inferred via parentage analysis. Text displays the *R* ^2^ value generated from a linear model (relative reproductive success ~ mating success) generated for females and males, respectively. The diameter of individual circles corresponds to the number of samples with a given value

The Bateman gradient was significantly positive in both sexes (Figure 4), with the number of mates strongly predicting relative reproductive success (females: *t* = 33.3, *p*‐value < 0.001; males: *t* = 53.1, *p*‐value < 0.001). The amount of variation in relative reproductive success explained by mating success was higher for males (*r* ^2^ = 0.80) than for females (*r* ^2^ = 0.59).

### 3.3 Sexual selection

**FIGURE 5:**
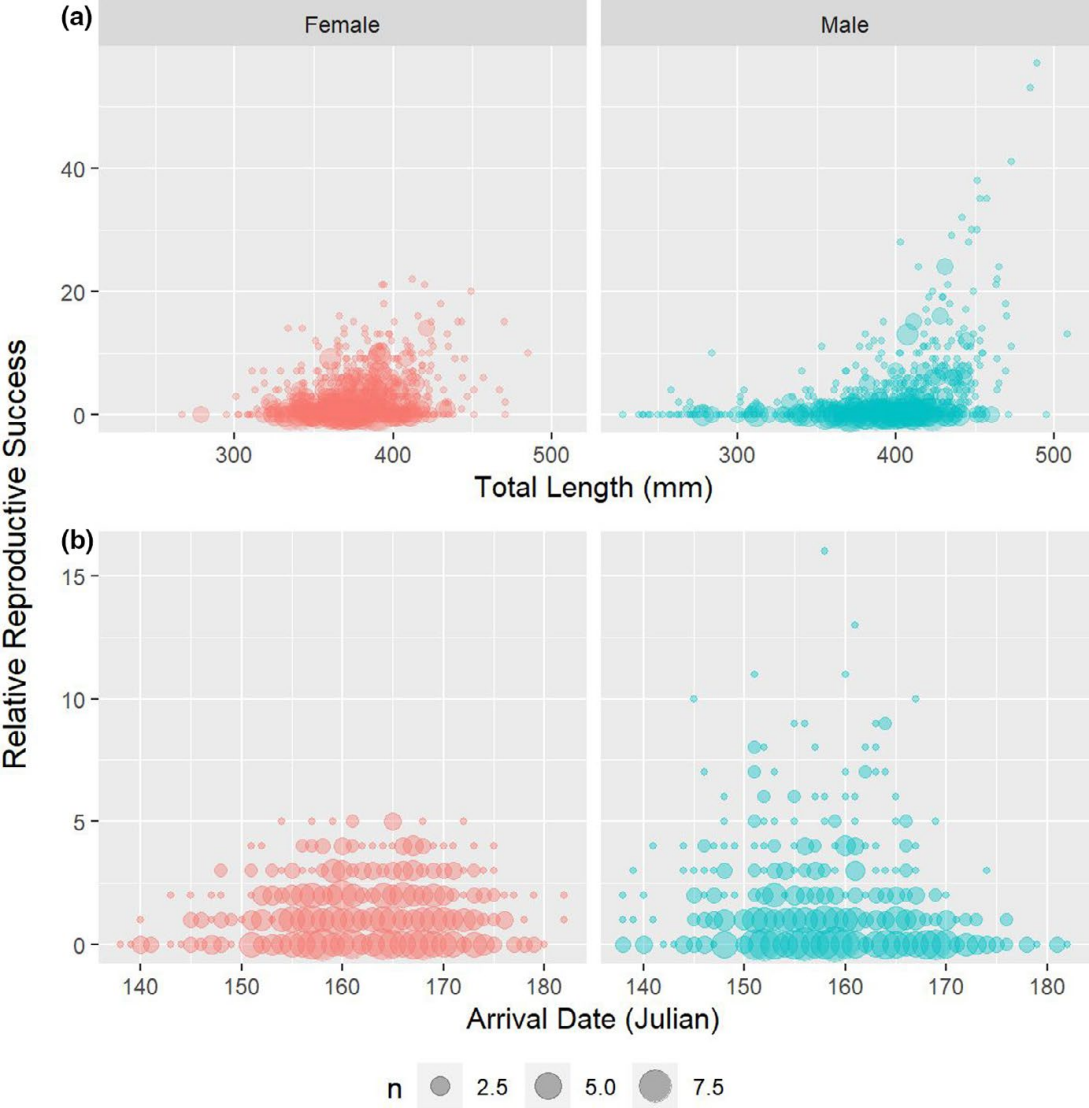
The relationship between (a) total length (mm), and (b) arrival date (Julian date) and relative reproductive success for male and female Yellowstone Cutthroat Trout (*Oncorhynchus clarkii bouvieri*). The size of circles is proportional to the number of observations for the specified value of total length/arrival date

The arrival date of fish that successfully mated was significantly earlier than unmated fish for males (*χ* ^2^ = 15.98, *df* = 1, *p*‐value <0.001), but not for females (*χ* ^2^ = 0.09 *df* = 1, *p*‐value = 0.77; Figure 5). Males which acquired mates arrived at the spawning grounds 3 days earlier (mean Julian date *µ* = 157) than those that failed to mate (*µ* = 160). There was a positive but nonsignificant relationship between mating success and arrival date for females (Pearson's *r* = 0.05, *t* = 1.5405, *df* = 774, *p*‐value = 0.124). The same relationship was significantly negative for males (Pearson's *r* = −0.12, *t* = −3.1389, *df* = 717, *p*‐value = 0.002). The impacts of arrival date on relative reproductive success were significantly negative for males (Pearson's *r* = −0.11, *t* = −2.8835, *df* = 717, *p*‐value = 0.004) but not females (Pearson's *r* = −0.01, *t* = −0.30463, *df* = 774, *p*‐value = 0.7607). Adults that successfully acquired mate(s) were significantly longer (*µ* ± *SE*: females = 381 ± 1.27 mm, males = 404 ± 2.06 mm) than unmated adults (*µ* ± *SE*: females = 370 ± 1.78 mm, males = 376 ± 2.63 mm) for both males (*χ* ^2^ = 75.44, *df* = 1, *p*‐value < 0.001) and females (*χ* ^2^ = 19.18, *df* = 1, *p*‐value < 0.001; Figure 5). The relationship between mating success and total length was significantly positive for both females (Pearson's *r* = 0.28, *t* = 8.1322, *df* = 774, *p*‐value < 0.001) and males (Pearson's *r* = 0.41, *t* = 10.961, *df* = 717, *p*‐value < 0.001). Length was also positively correlated with relative reproductive success for both sexes (females: Pearson's *r* = 0.28, *t* = 8.2221, *df* = 774, *p*‐value < 0.001; males: Pearson's *r* = 0.38, *t* = 11.891, *df* = 717, *p*‐value < 0.001).

**FIGURE 6:**
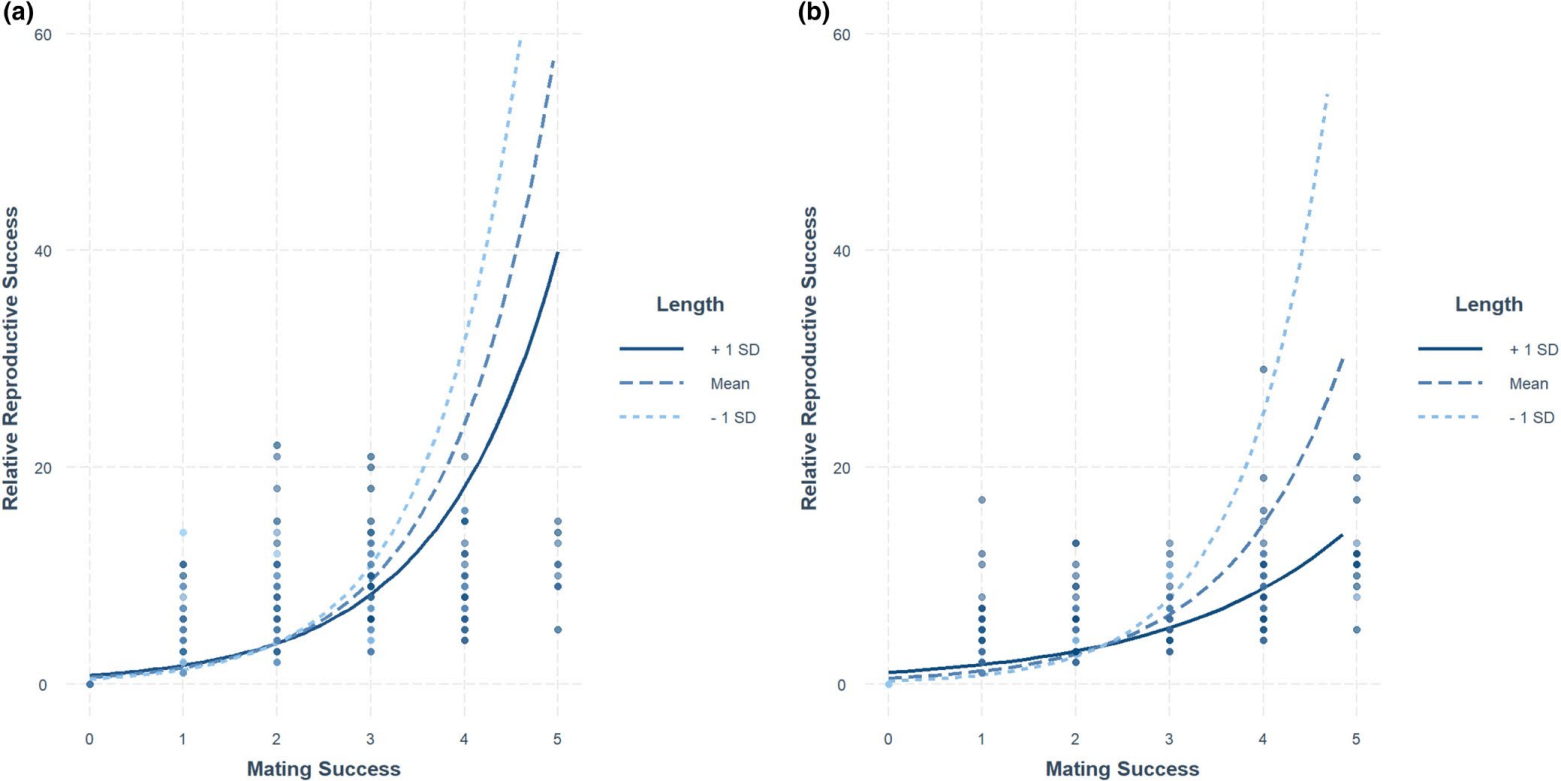
An interaction plot displaying the relationship between mating success, relative reproductive success, and total length for female (a) and male (b) adult Yellowstone Cutthroat Trout (*Oncorhynchus clarkii bouvieri*) sampled at an instream weir on Burns Creek, Idaho

The best‐supported model to explain the observed variation in relative reproductive success for both females and males included an interaction between mating success and total length (Table 2). For females, the interaction between mating success and length explained 19.3% of the observed variation in relative reproductive success, which was comparable (19.0%) to several other models which had a significantly lower model support (Akaike weight of top model, *w*_i_ = 0.982; all others ≤0.006). For females, each additional mate resulted in an increase of 2.67 offspring whereas each increase in length (1 mm) resulted in 0.009 additional offspring (Table 3). Similar to females, the top supported model was much more well supported (*w*_i_ = 1.0) than others (Table 3). Each additional mate obtained by a male resulted in an increase of 3.39 offspring, and increased length was positively related to reproductive success (each additional mm added 0.015 offspring). Model fit was higher for males (McFadden *R* ^2^ = 0.246) than for females (*R* ^2^ = 0.193). An interaction plot between mating success and length on relative reproductive success indicated that additional mates had a substantially greater impact on relative reproductive success for smaller adults relative to larger ones, and the strength of this relationship was greater for males than females (Figure 6).

**TABLE 2:** Outputs from generalized linear models which sought to explain the relative predictive importance of total length and arrival time at the spawning ground on relative reproductive success of Yellowstone Cutthroat Trout (*Oncorhynchus clarkii bouvieri*) in Burns Creek, Idaho

Sex	Model	AICc	∆AIC	*w*_i_	*K*	*R^2^*
Female	Mates * Length	2,770		0.982	5	0.193
	Mates + Date	2,780	10	0.006	5	0.190
	Mates * Date	2,780	10	0.006	5	0.190
	Mates + Length + Date	2,782	12	0.003	6	0.190
	Mates + Length * Date	2,783	13	0.001	8	0.190
	Mates	2,783	14	0.001	3	0.187
	Mates + Length	2,785	15	0.001	4	0.188
	Length + Date	3,363	593	0	5	0.019
	Length * Date	3,366	596	0	7	0.019
	Length	3,370	600	0	3	0.016
	Date	3,416	646	0	4	0.003
	Null	3,422	652	0	2	0.000
Male	Mates * Length	2,274	‐	1	5	0.246
	Mates + Length + Date	2,409	135	0	6	0.202
	Mates + Length * Date	2,411	137	0	8	0.203
	Mates + Length	2,413	139	0	4	0.200
	Mates + Date	2,424	150	0	5	0.197
	Mates * Date	2,424	150	0	5	0.197
	Mates	2,428	154	0	3	0.194
	Length + Date	2,840	566	0	5	0.058
	Length * Date	2,843	569	0	7	0.058
	Length	2,857	583	0	3	0.051
	Date	2,983	709	0	4	0.010
	Null	3,009	734	0	2	0.000

Note

Two suites of models were run, one each for male and female adults. Parameters are as follows: length = total length (mm), date = Julian date of arrival at an instream weir which was directly downstream from spawning habitats. AICc = Akaike information criteria corrected for small sample size; ∆AICc = difference between each model and the best performing model; and *w*_i_ = the Akaike weight; *K* = number of model parameters; *R* ^2^ = McFadden's pseudo‐*R* ^2^ which was used to evaluate model fit.

**TABLE 3:** Coefficients and standard errors for the effects of total length, mating success, and their interaction on reproductive success of male and female Yellowstone Cutthroat Trout (*Oncorhynchus clarkii bouvieri*) in Burns Creek, Idaho

Sex	Parameter	Estimate	*SE*	*Z*	*p*‐value
Female	Intercept	−3.89	0.84	−4.63	<0.001
	Mating success	2.67	0.39	6.85	<0.001
	Length	0.009	0.002	4.05	<0.001
	Mating success * Length	−0.005	0.001	−4.62	<0.001
Male	Intercept	−6.53	0.63	−10.37	<0.001
	Mating success	3.39	0.22	15.22	<0.001
	Length	0.015	0.002	9.73	<0.001
	Mating success * Length	−0.007	0.001	−12.88	<0.001

Note

This model was the best‐fit model based on model selection criteria the results of which are presented in Table 2.

### 3.4 Inbreeding avoidance among spawning adults

**FIGURE 7:**
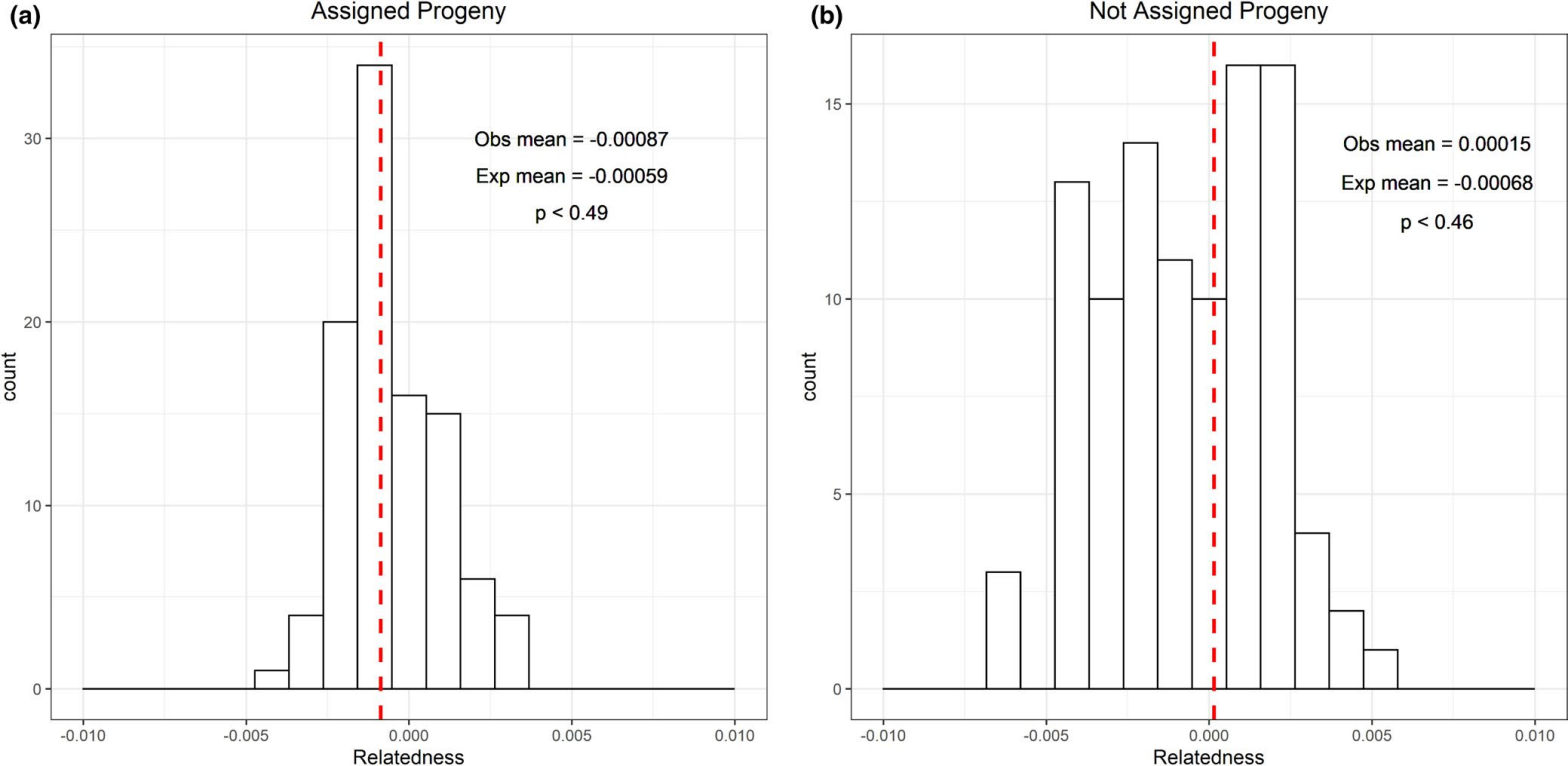
A histogram displaying expected values of relatedness (Rxy) among groups of adult Yellowstone Cutthroat trout (*Oncorhynchus clarkii bouvieri*): (a) adults assigned progeny via parentage analysis and (b) adults without any assigned progeny. Vertical dotted lines correspond to the observed mean levels of relatedness among individuals within a group (Obs mean) and mean expected values of relatedness (Exp mean) generated by randomly shuffling individuals between groups are listed in the upper right of each graph. *p*‐values represent the number of simulations out of the total number ran that were less than or equal to the observed value

Among the four potential relatedness estimators considered, the Queller and Goodnight (1989) coefficient displayed the highest correlation between observed and expected values of relatedness (Pearson's correlation coefficient *t* = 0.924). Observed relatedness among parents that were and were not assigned progeny was −0.00087 and 0.00015, respectively (Figure 7). Expected levels of relatedness among individuals that were and were not assigned progeny were −0.00059 (*SD*: 0.00260) and −0.00068 (0.00157), respectively (Figure 7). Neither value of observed relatedness within a group was significantly different than expected values (assigned progeny: *p* < 0.49 and not assigned progeny: *p* < 0.46).

### 3.5 Effective population size (*N*_e_) and number of breeders (*N*_b_)

The point estimate of *N*_e_ was 868 (787–959, 95% CI) and *N*_b_ was 576 (513–650, 95% CI). The ratio of *N*_b_/*N*_e_ was 0.66.

## 4 DISCUSSION

Using a parentage‐based tagging approach, we have provided the first description of the genetic mating system for Yellowstone Cutthroat Trout, a subspecies of conservation concern. While both the opportunity for selection and opportunity for sexual selection were more than twice as high for males than for females, only the variance in mating success (opportunity for selection) was statistically different between the two sexes. The Bateman gradients for females and males were both significantly positive; however, a greater proportion of reproductive success was explained by mating success for males (*r* ^2^ = 0.80) than females (*r* ^2^ = 0.59). The observed patterns of mating success, reproductive success, and their associated relationships indicated this population of Yellowstone Cutthroat Trout displayed a polygynandrous mating system, whereby both sexes experienced variation in mating success due to multiple mating, and sexual selection was variable across sexes (Avise et al., 2002; Jones & Avise, 2001). We detected evidence of sexual selection acting on total length, as mean estimates of mating success differed at these traits for mated and unmated Yellowstone Cutthroat Trout. We indirectly quantified sexual selection through generalized linear models, which confirmed the importance of length in predicting relative reproductive success, but arrival date was not in the best‐supported model. While we failed to detect a signal of inbreeding avoidance among adults, the group of parents that produced progeny were on average slightly less related than adults that did not produce progeny. Lastly, the effective number of breeders was lower than effective population size. These two parameter estimates had nonoverlapping confidence intervals which indicated that reproductive contributions in 2016 were less than the long‐term average and could be explained by several different factors (e.g., skip‐spawning, large variance in reproductive success, skewed operational sex ratio). Combined, the current study helps to fill several fundamental gaps in knowledge regarding the reproductive characteristics and mating system of Yellowstone Cutthroat Trout.

Social mating systems and spawning behaviors are well studied within some salmonids (see Esteve, 2005 for review); however, taxa remain for which there is little or no information, including Cutthroat Trout. Several traits observed in our study of Yellowstone Cutthroat Trout compare with patterns described for other members of the Salmonidae family. For example, we found evidence that individuals of both sex participated in polygamous (polygyny and polyandry) or monogamous matings, an observation previously noted for Steelhead (Seamons et al., 2004a), Brown Trout (*Salmo trutta*, Serbezov et al., 2010), and Brook Trout (Kanno et al., 2011). In Chinook Salmon (*O. tshawytscha*) and Atlantic Salmon, there is evidence of polyandry but not polygyny (Bentzen et al., 2001; Fleming, 1998). Theoretical expectations are that polygamy would be common among salmonids because one sex is free of parental care (males), and intrasexual selection in the form of male–male competition is strong (de Gaudemar, 1998). As a result, observing polygynous, polyandrous, and monogamous matings between individual Yellowstone Cutthroat Trout was not unexpected.

From a conservation and management perspective, a description of genetic mating systems is important as populations exposed to strong sexual selection and reproductive exclusion can experience a reduction in *N*_e_, as only a subset of the available adults will contribute to future generations (Waite & Parker, 1997). For the population of Yellowstone Cutthroat Trout in our study, we observed a population‐level polygynandrous mating system with both sexes exhibiting evidence of multiple mating. Multiple paternity can increase effective population size (*N*_e_) relative to systems with strict monogamy (Pearse & Anderson, 2009; Waite & Parker, 1997); however, this is directly influenced by the strength of sexual selection and type of mating system. We observed multiple paternity with males mating with multiple females and vice versa and argue that if multiple paternity came at the cost of reproductive contributions from unmated males, then *N*_e_ in our scenario would have been lower than a population of the same size exhibiting strict monogamy. Nonetheless, mating system insights gleaned from this study illustrate several important points; namely, males are subject to stronger sexual selection than females, and male total length interacted with mating success to predict relative reproductive success.

Another trait observed in Yellowstone Cutthroat Trout that mirrors observations from other salmonids was protandry, whereby males arrived earlier than females to spawning grounds. Protandry has been widely documented in Pacific salmon (Morbey, 2000) and Steelhead (McMillan et al., 2007, although see Seamons et al., 2004b), and it has been hypothesized that males arrive early to establish dominant access to spawning females (e.g., Healey & Prince, 1998). The ability for males to establish dominance over competing males can be influenced by body size (Dickerson et al., 2002; Fleming, 1998; Quinn & Foote, 1994), prior residence (Foote, 1990), and the ratio of sexually active females to males (e.g., operational sex ratio; OSR). In Burns Creek, successfully mated males arrived earlier on average to the spawning grounds than males that failed to mate. Furthermore, later arrival dates were correlated with decreased mating success and relative reproductive success, but arrival date was not in the best‐fit model that explained relative reproductive success. These observations suggest that while protandry may be advantageous for individual males, at the population‐level, size may be a much more important factor in predicting mating success.

There are numerous examples in the salmon literature of reproductive success being positively related to body size (e.g., Anderson et al., 2013; Berejikian et al., 2009; Fleming, 1996; Fleming & Gross, 1992; Quinn & Foote, 1994; Schroder, 1981; Seamons et al., 2004b); however, there are other instances where the relationship is either weak, nonexistent, or affected by other forms of selection (e.g., Dickerson et al., 2005; Seamons et al., 2004b, 2007). Additionally, work in steelhead identified factors such as length and arrival date that can vary in their impact depending on spawn year (Seamons et al., 2004b). Important to note, different life‐history strategies (e.g., precocial males and jacks), behavioral strategies (e.g., sneaker or satellite males), and other forms of selection (e.g., predation by terrestrial animals) may alter the strength of the relationship between arrival time and body size on reproductive success (Dickerson et al., 2002, 2005; McMillan et al., 2007). In our system, fish that successfully mated were longer than those that did not, but whether this trend is consistent across years remains unknown. Our evaluation of mating success predictors failed to incorporate environmental variables which may have also influenced the distribution of mating and reproductive success (e.g., Quinn et al., 2001). Lastly, although we made concerted efforts to cull sexually immature fish from our analyses of mating systems and sexual selection, it is possible that some fish which were incapable of mating remained, thus introducing bias in our estimates. Future studies should consider monitoring across multiple spawn years to determine the consistency of trends observed here and identify potentially important environmental variables.

Parentage analysis identified offspring parent pair relationships (trio) for ~79% of the sampled offspring, which indicates a fraction of contributing parents were not sampled. The presence of unsampled parents may have occurred as a result of either failing to sample migrating adults as they passed the weir or a failure to sample resident fish living above the weir which contributed to reproduction. We argue the latter scenario was the most likely as the weir is a permanent structure and blocks upstream passage. In PBT programs, the tagging rate, or proportion of offspring tagged via genetic sampling of adults, is equal to the percentage of sampled adult males times the percentage of sampled adult females (Steele et al., 2019). In our case, 78.7% of offspring were assigned which implies 88.7% of contributing adults (e.g., 0.887 males × 0.887 females = 0.787 of offspring assigned) contributing to reproduction in Burns Creek were sampled. This raises potential concerns about failing to capture all data on adult reproduction (e.g., patterns of protandry and adult size). If the 12% of contributing parents that were unsampled were resident, then our results apply to the migratory portion of the population. Based on our observed PBT assignments, the majority of adults contributing to reproduction in this system were migratory and are therefore drivers of persistence and population structure in the lower stretches of Burns Creek. If the migratory behavior of Yellowstone Cutthroat Trout is heritable, as it is in other salmonids (e.g., Pearse et al., 2014), then conserving this component of the population is most important for the management of mainstem fishery. Lastly, our failure to tag 100% of offspring highlights the difficulty with applying PBT in natural systems (as opposed to controlled environments such as hatcheries).

The parent pair–offspring relationships identified via PBT used to infer mating systems displayed a high level of concordance with results from sibship analysis. Our findings mirror previous efforts to resolve full‐sibling families via sibship methods (Ackerman et al., 2016), and highlight it is possible to accurately estimate sibship and *N*_b_ without parental genotypes (Waples & Waples, 2011). The ability to infer family structure using only offspring from a single generation is significant, as it can be applied in scenarios where sampling both parents and their offspring are either logistically difficult or not possible.

Pairwise genetic relatedness among adult Yellowstone Cutthroat Trout which mated was not significantly different relative to adults that failed to reproduce, and this trend has been noted for other salmonids. Several studies have identified mate choice among salmonids, specifically at loci associated with the major histocompatibility class (MHC). In particular, both Atlantic Salmon and Chinook Salmon select mates to increase heterozygosity at the MHC loci, but not at putatively neutral loci (Landry& Bernatchez, 2001; Neff et al., 2008). Because we only surveyed Yellowstone Cutthroat Trout at putatively neutral SNP loci, we were unable to test for mate association at MHC, but nonetheless, patterns of neutral genetic relatedness among parents supported a random mating scheme.

Lastly, we generated estimates of *N*_b_ and *N*_e_ and demonstrated the *N*_b_/*N*_e_ was less than one for Yellowstone Cutthroat Trout. Ratios of *N*_b_/*N*_e_ vary across freshwater fish (Brown Trout: 0.48, Lake Trout, *Salvelinus namaycush*: 1.212, Razorback Sucker, *Xyrauchen texanus*: 1.004) and simple life‐history characteristics such as age at maturity and adult lifespan can explain up to 2/3 of the variation in observed *N*_b_/*N*_e_ ratios (Waples et al., 2013). In the case of Yellowstone Cutthroat Trout in the South Fork Snake River, there are two pools of potential parents capable of contributing to annual reproduction: tributary residents and mainstem adults which perform fluvial spawning migrations (Thurow et al., 1988). The fact that we observed *N*_b_/*N*_e_ ratios less than one could be explained by only a subset of the potential parent population contributing to reproduction (i.e., reduced resident or fluvial contributions or skip‐spawning), an unusually large variance in reproductive success, or a highly skewed operational sex ratio that occurred in 2016 relative to other spawn years. Additionally, estimation of *N*_e_ involved an assumption of random mating, and mating success was not random in this population, implying potential bias in our estimate of effective population size. Because we only have data for a singular spawn year, it is difficult to identify specific mechanisms to explain our observed *N*_b_/*N*_e_ ratio, but nonetheless, both these values are sufficiently high to suggest Yellowstone Cutthroat Trout in Burns Creek represent a genetically stable and diverse population.

In conclusion, we used data from a single spawn year to describe the genetic mating system and identify predictors of reproductive success for a population of migratory Yellowstone Cutthroat Trout in Burns Creek, Idaho. Moving forward, these data can serve as a baseline for future monitoring efforts and a template for investigations into the reproductive ecology of additional populations of Yellowstone Cutthroat Trout and other freshwater salmonids. With respect to preserving the genetic mating system and reproductive outputs, maintaining spawning habitats, their connectivity to the mainstem rivers, as well as protection of the breeding population are of utmost importance (Muhlfeld et al., 2019; Shepard et al., 2019). For example, the observation that the largest adults were responsible for producing a disproportionate amount of offspring may serve as a basis for angling restrictions. Gwinn et al. (2015) identified protection of the largest adults in the population via harvest slots (i.e., harvest is restricted to fish of intermediate length) consistently produced greater numbers of fish harvested and greater catches of trophy fish while conserving reproductive biomass and a more natural population age structure. Throughout much of their range, the harvest of Yellowstone Cutthroat Trout is prohibited, but in areas where harvest is still allowed or is being considered, data generated here may be of direct relevance.

## CONFLICT OF INTEREST

The authors report no conflict of interest.

## AUTHOR CONTRIBUTIONS

**John S. Hargrove:** Conceptualization (equal); Formal analysis (equal); Methodology (lead); Visualization (lead); Writing‐original draft (lead); Writing‐review & editing (lead). **Jesse McCane:** Data curation (lead); Formal analysis (supporting); Validation (lead); Writing‐review & editing (equal). **Curtis J. Roth:** Conceptualization (equal); Data curation (supporting); Formal analysis (supporting); Investigation (lead); Methodology (lead); Project administration (equal); Writing‐original draft (supporting); Writing‐review & editing (equal). **Brett High:** Conceptualization (equal); Investigation (equal); Methodology (equal); Project administration (equal); Resources (equal); Writing‐review & editing (equal). **Matthew R. Campbell:** Conceptualization (lead); Funding acquisition (lead); Resources (lead); Supervision (lead); Writing‐original draft (supporting); Writing‐review & editing (equal).

## Data Availability

Individual genotypes and size data are archived on Dryad (https://doi.org/10.5061/dryad.zcrjdfncd).
